# Protective Activity of Melatonin Combinations and Melatonin‐Based Hybrid Molecules in Neurodegenerative Diseases

**DOI:** 10.1111/jpi.70008

**Published:** 2024-11-25

**Authors:** Francesca Galvani, Mariarosaria Cammarota, Federica Vacondio, Silvia Rivara, Francesca Boscia

**Affiliations:** ^1^ Department of Food and Drug University of Parma Parma Italy; ^2^ Division of Pharmacology, Department of Neuroscience, Reproductive Sciences and Dentistry, School of Medicine Federico II University of Naples Naples Italy

**Keywords:** antioxidant activity, drug combination, hybrid compounds, melatonin, neuroprotection, polypharmacology, synergistic activity

## Abstract

The identification of protective agents for the treatment of neurodegenerative diseases is the mainstay therapeutic goal to modify the disease course and arrest the irreversible disability progression. Pharmacological therapies synergistically targeting multiple pathogenic pathways, including oxidative stress, mitochondrial dysfunction, and inflammation, are prime candidates for neuroprotection. Combination or synergistic therapy with melatonin, whose decline correlates with altered sleep/wake cycle and impaired glymphatic “waste clearance” system in neurodegenerative diseases, has a great therapeutic potential to treat inflammatory neurodegenerative states. Despite the protective outcomes observed in preclinical studies, mild or poor outcomes were observed in clinical settings, suggesting that melatonin combinations promoting synergistic actions at appropriate doses might be more suitable to treat multifactorial neurodegenerative disorders. In this review, we first summarize the key melatonin actions and pathways contributing to cell protection and its therapeutic implication in Alzheimer's disease (AD), Parkinson's disease (PD), and multiple sclerosis (MS). We remark the major controversies in the field, mostly generated by the lack of a common consensus for the optimal dosing, molecular targets, and toxicity. Then, we review the literature investigating the efficacy of melatonin combinations with approved or investigational neuroprotective agents and of melatonin‐containing hybrid molecules, both in vitro and in animal models of AD, PD, and MS, as well as the efficacy of add‐on melatonin in clinical settings. We highlight the rationale for such melatonin combinations with a focus on the comparison with single‐agent treatment and on the assays in which an additive or a synergistic effect has been achieved. We conclude that a better characterization of the mechanisms underlying such melatonin synergistic actions under neuroinflammation at appropriate doses needs to be tackled to advance successful clinical translation of neuroprotective melatonin combination therapies or melatonin‐based hybrid molecules.

**Abbreviations**
AChE: acetylcholinesterase
AD: Alzheimer's disease
AMPK: AMP‐activated protein kinase
α‐Syn: α‐synuclein
βAPP: beta amyloid precursor protein
BuChE: butyrylcholinesterase
DADA: diisopropylamine dichloroacetate
DAPK: death‐associated protein kinase 1
EAE: experimental autoimmune encephalopathy
FAAH: fatty acid amide hydrolase
GSH: glutathione
GSSG: glutathione disulfide
LPS: lipopolysaccharide
MCI: mild cognitive impairment
MPTP: 1‐methyl‐4‐phenyl‐1,2,3,6‐tetrahydropyridine
MS: multiple sclerosis
NOS: nitric oxide synthase
Nrf2: nuclear factor erythroid 2‐related factor 2
PAS: periferic anionic site; PBMC; peripheral blood mononuclear cell
PD: Parkinson's disease
PPAR: peroxisome proliferator‐activated receptor
PPMS: primary progressive multiple sclerosis
ROS: reactive oxygen species
RRMS: relapsing‐remitting multiple sclerosis
SPMS: secondary progressive multiple sclerosis

## 1 Introduction

Neurodegenerative disorders are recognized as a major cause of disability and death worldwide, representing a huge global health burden with an increasing incidence rate [1, 2]. Despite the efforts of the scientific community to deepen knowledge of the etiology and pathogenetic mechanisms and develop new pharmacological agents or identify effective combinations of available drugs, to date, no treatment is available to prevent or halt the neurodegenerative process. Approved drugs able to slow down disease progression, mainly acting purely symptomatic at early disease stages, represent the best‐case scenario for patients affected by neurodegenerative conditions [3]. Given the mechanisms of action [4] and the age‐related decline of physiological production, melatonin has been widely investigated in both preclinical and clinical settings in Alzheimer's disease (AD), Parkinson's disease (PD), and multiple sclerosis (MS) [5, 6, 7, 8, 9]. Despite favorable outcomes have been reported for several biological and behavioral markers of disease insurgence and progression in preclinical studies, mild or poor outcomes have been observed in clinical studies, although an improvement in patient sleep quality is reported following melatonin administration in all disease conditions, including AD, MS, and PD [10, 11, 12, 13]. Several reasons can be at the basis of the positive, yet not therapeutically exploitable activity of melatonin, including the limited efficacy of the molecule and the ability to counteract only some of the neurodegenerative processes and symptoms associated with these diseases. Therefore, combinations of melatonin with other neuroprotective agents have been investigated, pointing to a synergistic or additive effect in the context of a multitarget‐directed strategy often pursued in neurodegenerative diseases characterized by a multifactorial nature. Two kinds of combinations were investigated: (i) combination of melatonin with neuroprotective agents, either approved drugs or substances with mechanisms supporting a neuroprotective effect (e.g., antioxidant, metal chelator, etc.); (ii) melatonin‐based hybrid compounds merging melatonin structure with that of other antioxidant and neuroprotective molecules. Both approaches offer potential advantages and have inherent limitations. Drug combinations allow to modify the dosage of each agent to optimize efficacy and limit toxicity. On the other hand, the pharmacological interactions deriving from drug combination might produce amplified adverse reactions and decrease the effectiveness of combined molecules with redundant effects. These drawbacks could be mitigated using a single multifunctional compound, acting on two or more targets at the same time, for which drug‐like properties can be optimized. Compared to drug combinations, these compounds may offer the advantage of reduced side effects and pharmacokinetic complexity, and fewer drug−drug interactions. The decrease in treatment complexity can ameliorate patient compliance, resulting into an improved management of the disease. Notably, multitarget compounds could suffer from a challenging optimization of physicochemical properties and pharmacokinetic profiles. Additionally, the inclusion of the pharmacophore portions engaging each target in a fixed ratio could pose problems in the determination of the effective and safe dose, especially when the active concentrations at the two or more targets are significantly different [14, 15].

In the present review, we first briefly discuss the protective functions and mechanisms of melatonin‐mediated cell protection. We also mention the major controversies related to the use of melatonin as a neuroprotective agent, mostly generated by the lack of a common consensus for the optimal dosing, molecular targets, and toxicity. Then, we summarize the preclinical studies investigating the efficacy of melatonin as well as melatonin combinations with other agents and of melatonin‐containing hybrid molecules both in vitro and in animal models of AD, PD, and MS. The use and efficacy of melatonin as an add‐on to patient standard therapy is reported. A description of the rationale for the molecular combination, the experimental models and the results is provided, focusing on the comparison with single‐agent treatment and on the assays in which an additive or a synergistic effect was achieved.

## 2 Mechanisms of Melatonin‐Mediated Cell Protection

Melatonin neuroprotective activities include cell protection from oxidative stress, regulation of energy metabolism, and modulation of the innate immune system. It is reported that melatonin exerts neuroprotective actions both directly on neuronal cells and indirectly through the modulation of glial cell activities [16, 17]. A considerable number of controversies persist in the field among scientists [18, 19], mostly generated by a plethora of studies describing the impressive beneficial actions of melatonin in diverse pathological conditions without a common consensus for the optimal dosing and defined molecular targets. As recently discussed by Boutin and Jockers [19], in most of the studies displaying protective effects, melatonin is used at supraphysiological or pharmacological concentrations (beyond 1 µM, and up to several mMs), far above the physiological levels (1 pM to 100 nM). Such high concentrations are difficult to reach in patients after oral treatment due to the low bioavailability reported in clinical trials (ranging from 10% to 56%), also due to an extensive first pass metabolism [20, 21]. It is reported that 1−3 mg melatonin treatment in vivo results in a *C*_max_ ~ 10 nM, while given at 100 mg dose melatonin produces a circulating concentration of about 0.5 µM and has a short half‐life (T_1/2_ ~ 50 min), both after oral or intravenous administration [22]. Additionally, the determination of the effects resulting from melatonin metabolites [23], such as 6‐hydroxymelatonin [24], requires further investigations. Moreover, at pharmacological concentrations, the full spectrum of targets and mechanisms by which melatonin acts on cells, including brain cells, needs a deeper investigation and validation. Indeed, it is still debating whether the pineal hormone actions at such high concentrations involve membrane or nuclear receptors, receptor‐independent processes, or multiple mechanisms participate simultaneously [19, 25].

The role of melatonin MT_1_ and MT_2_ G‐protein‐coupled receptors in regulating physiological processes such as circadian rhythms, sleep, seasonal reproduction, immune functions, retinal physiology, and glucose homeostasis has been well characterized (reviewed in [26, 27, 28]). Under pathological conditions, some melatonin effects may persist even in the absence of MT_1_ and MT_2_ receptors or upon their pharmacological blockade, indicating the existence of melatonin receptor‐independent mechanisms, as observed in studies evaluating the neuroprotective activity of melatonin [29]. Moreover, in vitro studies performed in transfected HEK293 cells have shown that melatonin receptors may be desensitized by internalization when exposed beyond 1 µM melatonin, suggesting that non‐receptor‐dependent mechanisms or indole‐compound‐based actions may be relevant when high concentrations of melatonin are given [30, 31]. Nevertheless, it cannot still be excluded that some functions of melatonin at pharmacological concentrations (1 μM and above) might be explained by its ability to inhibit the MT_3_ receptor, a quinone reductase 2 enzyme (NQO2) (IC_50_ in the 10−100 μM range) [32]. The development of selective melatonin receptor ligands, the characterization of melatonin receptor‐selective KO mice, and the identification of the phenotype of brain cells expressing melatonin receptors under pathological conditions using well‐characterized antibodies (for discussion see [33]) will be instrumental to establish and validate the role of melatonin receptors in neuroprotection. Here, we briefly describe the major actions attributed to melatonin and the controversies in each field.

### 2.1 Protection From Oxidative Stress

Accumulating evidence has shown that melatonin alleviates the pathological injury caused by oxidative stress, including lipid peroxidation and DNA damage. It is reported that melatonin antioxidant mechanisms involve free radical scavenging and metal chelation activity, stimulation of antioxidative defensive systems, and reduction of mitochondrial oxidative stress. Such actions are not mechanistically well defined but reported to contribute to the anti‐apoptotic and anti‐inflammatory activities exerted by melatonin.

#### 2.1.1 Free Radical Scavenging and Metal Chelating Properties

A scavenger is a molecule that physically traps oxygen, nitrogen, or sulfur‐centered radicals [34]. At supraphysiological concentrations, within the micromolar‐millimolar range, the melatonin's ability to scavenge a wide variety of radicals in acellular systems is undoubted [35, 36, 37, 38, 39, 40, 41]. Due to the electron‐rich aromatic indole ring, melatonin and its metabolites are potent electron donors and decrease reactive oxygen and nitrogen species (ROS/RNS) as well as lipid peroxyl radicals in different models of biological membranes including rat brain or liver homogenates (at 5 mM) [42, 43] or in 2,2′‐azobis(amidinopropane) dihydrochloride (AAPH)‐induced human erythrocyte hemolysis (at 3−1000 µM) [44]. A number of studies in acellular systems or models of biological membranes have also reported the ability of micromolar‐millimolar melatonin in preventing metal‐induced free radical generation due to its metal chelating ability [45, 46, 47]. However, there is no general consensus that melatonin scavenger action occurs in vivo, in more complex living (animal) systems [19, 48]. The current criticism is generated by the observation that no scavenger really exists in a living context, as clearly described by Forman's seminal papers [34, 49], given the huge concentrations that would be needed to obtain an effective activity and the extremely short‐living nature of radical species. Therefore, a molecule with antioxidant properties in vivo can be considered as an inductor of natural enzymatic defenses.

#### 2.1.2 Stimulation of Antioxidative Defensive Systems

Melatonin has been described as an antioxidant molecule in cellulo, since it is able to induce cellular enzymatic antioxidant defenses through a mechanism of action that still needs to be completely understood and validated. At pharmacological concentrations, melatonin is able not only to inhibit the activity of the pro‐oxidative enzyme quinone reductase 2, formerly described as the MT_3_ binding site [50] but also to increase the expression and/or activity of antioxidant enzymes in different disease contexts, including AD and MS [51, 52, 53]. Recently, Monteiro et al. [54] reviewed the studies assessing the effects of melatonin on glutathione peroxidase, glutathione reductase, superoxide dismutase, and catalase antioxidant enzymes in different experimental animal models of tissue damage in rats, including neurological diseases. In these studies, melatonin was used at pharmacological doses from 0.1 to 20 mg/kg/day (i.p. or orally). However, there is still no experimental evidence of melatonin direct binding to response elements in antioxidant enzymes or to nuclear factors promoting the expression of the defense enzymes. In several disease contexts, including neurodegenerative disease, the activation of MT_1_ or MT_2_ receptor signaling and the translocation and upregulation of the antioxidative nuclear factor erythroid 2 (Nrf2) mediate these effects. The MT_2_‐selective antagonist 4‐phenyl‐2‐propionamidotetralin (4‐P‐PDOT) was able to prevent the activation of the Nrf2 pathway and the neuroprotective actions afforded by 1 μM melatonin in vitro, against erastin‐induced ferroptosis in neuronal hippocampal HT‐22 cells, and by 10 mg/kg melatonin (i.p.), in vivo, against the spinal cord injury [55, 56].

#### 2.1.3 Reduction of Mitochondrial Oxidative Stress

Mitochondria are the primary site for ROS generation and are the major targets of melatonin protection from oxidative damage. There is constant debating on intra‐mitochondrial melatonin synthesis [57, 58, 59]. Suofu et al. [59] demonstrated that nonsynaptosomal brain mitochondria can indeed synthesize melatonin if the serotonin precursor is available. However, the experimental evidence for melatonin synthesis in all cells is still considered weak, thus remaining an important goal for future studies in pure mitochondria. Moreover, the specific mechanisms by which melatonin limits oxidative damage to this organelle remain to be defined. Accumulating evidence has linked the upregulation of the silent information regulator sirtuin 3 (SIRT3), a major mitochondrial NAD+‐dependent histone deacetylase, to the ability of melatonin to protect mitochondria from oxidative stress. SIRT3 regulates the mitochondrial function via the deacetylation and consequent activation of key antioxidant enzymes such as the superoxide dismutase 2 (SOD2) [60]. In several disease contexts, including cerebral ischemia, pharmacological doses of melatonin ranging from 10 to 30 mg/kg i.p. in vivo or 25 μM−1 mM in vitro alleviated mitochondrial impairments through SIRT3 upregulation and SOD2 deacetylation; conversely, SIRT3 knocking down, knocking out or its pharmacological blockade significantly prevented melatonin protective actions [61, 62, 63, 64, 65, 66, 67, 68]. Suofu et al. [59] proposed that melatonin stimulation of the MT_1_ mitochondrial receptor regulates mitochondrial deacetylation processes via SIRT3. In human liver, L‐02 cells exposed to sodium fluoride and 40 μM melatonin, MT_1_ receptors silencing or MT_1_ receptors blockade with luzindole significantly prevented SIRT3‐mediated SOD2 activation [61].

### 2.2 Regulation of Energy Metabolism

Melatonin influences and maintains mitochondrial metabolism and redox homeostasis [69]. Melatonin regulates the concentration of acetyl‐CoA [70] and stimulates the activity of respiratory chain enzymes, oxidative phosphorylation, and ATP production [71, 72]. Melatonin limits electron leakage from the mitochondrial respiratory chain and supports mitochondrial biogenesis and fusion [73]. Mitochondrial melatonin effects are relevant under pathological conditions. To reduce mitochondrial oxidative stress, melatonin stimulation of MT_1_ receptor on mitochondrial outer membranes inhibits stress‐mediated cytochrome C release, caspase activation, and apoptosis [59]. Melatonin regulation of anti‐apoptotic proteins, mitophagy, and autophagy contributes to the protective effects in a range of pathological conditions [74]. Melatonin mitochondrial effects can be mediated by the activation of membrane receptors, of SIRT1 and SIRT3 pathways, and downstream signaling molecules such as peroxisome proliferator‐initiated receptor gamma coactivator 1‐alpha (PGC1α), adenosine 5'‐monophosphate‐activated protein kinase (AMPK), and Forkhead box protein O1 (FOXO1) [75, 76, 77]. These protective mechanisms were observed in vitro, under cadmium‐induced hepatotoxicity, with 1 μM melatonin concentration, and in vivo, under isoproterenol‐induced myocardial injury or cerebral ischemia/reperfusion (I/R) injury, at a minimum effective dose of 10 mg/kg i.p. [64, 65].

### 2.3 Modulation of the Innate Immune System

Melatonin is an immune modulator enhancing both defense mechanisms, which include proinflammatory processes observed under basal conditions and anti‐inflammatory responses in high‐grade inflammation [78, 79]. Controversy among studies is whether melatonin may be considered an immune‐defense inducer able to enhance immune system functions, or an anti‐inflammatory adjuvant able to stimulate anti‐inflammatory signaling with consistent effect in humans during pathological situations. A very recent study performed by Boutin et al. [80] showed that melatonin has no capacity, up to 10 μM concentration, to significantly influence: (a) ROS production in human peripheral blood mononuclear cells (PBMC) exposed to H_2_O_2_; (b) the release of proinflammatory (Il‐17, Il‐1b, IFN‐γ) or anti‐inflammatory (IL‐10, IL‐4) cytokines in PBMC or T cells stimulated with anti‐CD3 and anti‐CD28 antibodies or lipopolysaccharide (LPS); (c) phagocytosis from PMNs; (d) immunoglobulins release from B cells under IL‐21 exposure. Conversely, accumulating evidence in the last decade supported the ability of melatonin to stimulate anti‐inflammatory signaling in peripheral monocyte‐derived macrophages and brain‐resident microglia. Indeed, under LPS exposure, 10 nM melatonin reduced pyrin domain‐containing protein 3 (NLRP3) inflammasome activation in BV2 microglia [81]. At 1 mM concentration, melatonin inhibited LPS‐induced chemokine expression and secretion in BV2 cells [82], reduced cyclooxygenase‐2 expression in murine macrophage cell line RAW 264.7 [83, 84], downregulated the inducible NOS M1 marker, and upregulated the anti‐inflammatory M2 markers arginase 1 (Arg1) and the mannose receptor CD206 through signal transducer and activator of transcription 3 (STAT3) phosphorylation in primary microglia cultures [85]. In LPS‐treated animals, significant inhibition of the expression of proinflammatory mediators in microglia and NLRP3 inflammasome activation was observed when melatonin was given intraperitoneally at 10 mg/kg [83] or 30 mg/kg [86]. Although further studies are required to clarify the specific mechanism, several studies have linked this melatonin action to the activation of membrane receptors and stimulation of SIRT1‐associated pathways [87, 88]. SIRT1 is a nuclear NAD+‐dependent histone deacetylase that reduces the acetylated forms of several proinflammatory proteins, including the p65 subunit of the nuclear factor kappa B (NF‐κB). Moreover, several additional biological pathways have been shown to sustain the beneficial action of melatonin under inflammatory conditions, including the upregulation of the antioxidant Nrf2‐pathway, the downregulation of proinflammatory microRNAs, and exosome release [89, 90].

## 3 Neurodegenerative Diseases

AD, PD, and MS are diseases of the CNS lacking a cure and leading to a progressive loss of cognitive and/or motor functions. AD and PD are the most prevalent neurodegenerative diseases in the aged, while MS is the most common cause of neurological disability in young adults [91, 92, 93]. AD and PD are proteinopathies, as they are associated with the intracellular accumulation of toxic protein aggregates. The Amyloid‐β (Aβ)‐positive plaques, tau‐positive neurofibrillary tangles, biometal dyshomeostasis, cholinergic neuron degeneration, and a progressive cognitive impairment define AD [91]. The neuronal deposition of α‐synuclein (α‐syn, Lew bodies), the loss of dopaminergic neurons in the *substantia nigra*, and the progressive deterioration of motor functions are associated with PD [92]. The development of focal inflammatory and demyelinating lesions in white and deep gray matters due to T‐lymphocytic and macrophage infiltrations are the predominant features of MS leading to oligodendrocyte and axonal dysfunction. Clinically, the loss of motor function is the most visible symptom of MS; cognitive deficits often emerge early in the disease, but impairment is more prevalent during the progressive stage [94]. Additionally, a reduced volume of the pineal gland was observed in female MS patients [95].

Despite the different etiologies (i.e., infections, genetic mutations, protein aggregations) and clinical aspects, neuronal damage in all these diseases is associated with oxidative stress, mitochondrial dysfunction, and chronic activation of an innate immune response [96, 97].

The disruption of circadian rhythm and sleep/wake cycle is a shared symptom of such diseases and is accompanied by an impairment of the glymphatic CNS “waste clearance” system, leading to the accumulation of harmful proteins such as Aβ, tau, and possibly α‐syn to the brain parenchyma [98]. Sleep disturbances and glymphatic system deterioration intensify each other, ultimately contributing to neuroinflammation, and accelerating the progression of neurodegeneration [99, 100]. Pineal melatonin, whose rhythmic secretion is driven by the circadian clock in the suprachiasmatic nucleus of the hypothalamus, has a major role in the synchronization of the sleep/wake cycle. It is secreted at nighttime into the circulatory system and cerebrospinal fluid and has direct access to the brain and spinal cord through the glymphatic system [101, 102, 103]. Although direct scientific evidence linking the impairment of the glymphatic system, sleep disturbance, and altered melatonin secretion in neurodegenerative diseases is still lacking, it was shown that melatonin treatment (10 µg/g body i.p.) restored the glymphatic system function and circadian rhythmicity polarization of aquaporin‐4 water channels in astrocytes via alleviating sleep‐wake rhythm disruption and rectifying the abnormal expression of the circadian rhythm proteins Per2, Bmal1, Clock, and Per1 in a mouse model of depression [104].

### 3.1 Melatonin Decline in AD, PD, and MS

The nocturnal decline of the melatonin peak is associated with sleep disorders and disease progression [105]. The reduced melatonin secretion due to pineal gland dysfunction with or without suprachiasmatic nucleus degeneration [106] leads to impaired or incomplete melatonin signaling in AD [107, 108], PD [109], and MS patients [110, 111]. Such dysfunction is also based on changes in brain receptor densities as a consequence of neurodegeneration or compensatory actions [112, 113]. Melatonin levels, whose production is modulated by seasonal variations in night length, negatively correlate with MS activity in humans [111]. Single nucleotide polymorphisms in the genes encoding tryptophan hydroxylase 2, catalyzing the first step of melatonin biosynthesis, and MT_2_ receptor are associated with an increased risk of progressive MS [114].

### 3.2 Therapeutic Implication of Melatonin‐Based Therapies in AD, PD, and MS

A number of preclinical studies describe the beneficial effects of pharmacological doses of melatonin to treat sleep disturbances and alleviate pathogenesis development in experimental models of AD, PD, and MS. Likely based on the over‐enthusiastic interpretations of some preclinical data, several clinical studies assessing the therapeutic potential of oral melatonin ranging from 0.5 to 100 mg/kg were performed in AD, PD, and MS patients. A relevant but often poorly considered aspect of melatonin treatment is related to the safety of the molecule. Melatonin is generally considered a drug with very low toxicity, even in specific conditions, such as pregnancy and breastfeeding, for which melatonin use is declared probably safe in humans [115]. Nevertheless, only a limited number of clinical trials investigated the potential adverse effects of exogenous melatonin as primary outcomes. Information on adverse events mostly derives from clinical trials examining the physiological and clinical effects of exogenous melatonin and assessing adverse effects as secondary outcomes, with variable quality of reporting [116]. In healthy volunteers, interactions with cardiovascular, reproductive, endocrine, and metabolic systems were recorded, which might be worsened in the presence of a disease condition [117]. Melatonin is described as a safe treatment in the case of short‐term use, even if adverse effects have been recorded, including headache, fatigue, dizziness, or daytime sleepiness. But very little is known about long‐term safety, for which specific studies are needed and assume great importance in the context of the treatment of neurodegenerative diseases. Additionally, the dose of melatonin administered should also be taken into consideration when evaluating drug toxicity, as high doses may increase the risk of adverse effects, as well as the time of administration may be also relevant [19, 118]. Besides the ethical and toxicity issues for long‐term studies performed with high melatonin doses, the overall mild or poor outcomes observed in these studies may suggest that the transposition of preclinical findings into human studies is difficult and the lack of efficacy can be attributed to several factors including the decline or desensitization of melatonin receptors or to the fact that pharmacological doses of melatonin cannot be reached in humans, or at least not for prolonged periods [118, 119, 120]. These observations further suggest that melatonin combinations or melatonin‐hybrid molecules might be indeed more appropriate in multifactorial neurodegenerative disorders, and may encourage more studies assessing the combination promoting synergistic actions at appropriate doses.

#### 3.2.1 AD

In vitro preclinical studies assessing the beneficial effects of melatonin on oxidative stress and cytotoxicity mediated by exposure to Aβ_1−40_, Aβ_1−42_, or Aβ_25−35_, tau aggregates, or okadaic acid were mostly performed using concentrations in the micromolar‐millimolar range, in a variety of models, including clonal lines of neurons (SH‐SY5Y, SK‐N‐BE(2), Neuro2A, PC12 cells) and microglia (BV2, HMC3 cells) [121, 122, 123, 124, 125, 126, 127, 128], rodent primary cultures of cortical neurons or microglia, as well as organotypic brain slices [125, 129, 130]. Within the nanomolar range, melatonin (100 nM) significantly inhibited the expression of β‐amyloid precursor protein (βAPP)‐secretase activity and preserved the functional integrity of Pin1, a prolyl isomerase regulating nonpathogenic conformations of both βAPP and tau in SH‐SY5Y cells [131]. Circular dichroism spectroscopy and transmission electron microscopy studies showed that 100 μM melatonin acts as a dissociation catalyst on misfolded Aβ protein aggregations both in the absence or presence of apolipoprotein E4‐containing particles [132]. The transposition of these latter findings in the human brain remains unclear.

A consistent number of in vivo preclinical studies assessed the beneficial effects of supraphysiological doses melatonin, in the range 0.15−80 mg/kg, on Aβ levels, oxidative stress, cholinergic activity, neuroinflammation and spatial learning and memory both in AD‐like (after exposure to Aβ_1−42_, AlCl_3_ + d‐galactose, streptozotocin), or AD experimental models (Tg2576 mice, APP/PS1 mice; 3xTg‐AD mice) [29, 51, 133, 134, 135, 136, 137, 138]. Melatonin treatment at 10 mg/kg, given daily for 1 month in 4‐month‐old 5×FAD mice, decreased the number of Aβ plaques, improved cognitive behavior, and increased the expression of proteins involved in the autophagy–lysosome pathway, mitophagy, and microglia phagocytosis, such as TREM2 [134]. Interestingly, 2 mg/mL melatonin, given in drinking water, augments the glymphatic Aβ clearance in Tg2576 mice [139]. As regards the mechanism for melatonin effect on decreasing accumulation of harmful proteins in AD, it has been shown that, in neuronal cells, 0.2−1 μM melatonin binds to death‐associated protein kinase 1 (DAPK1) and dose‐dependently promotes DAPK1 degradation via the ubiquitin‐mediated proteasome pathway, which results in increased activity of Pin1. This melatonin action was not prevented by the melatonin receptor antagonist luzindole [140].

Clinical trials assessing the effects of melatonin therapy on cognitive deficits and behavioral symptoms in AD patients were disappointed at all stages of the disease; positive outcomes on sleep parameters were observed only when melatonin was given short‐term at dosages from 3 to 5 mg in patients with mild AD [141]. At dosages from 3 to 24 mg, melatonin treatment benefitted sleep, cognition, and behavioral symptoms only in patients with mild cognitive impairment (MCI) regardless of the treatment duration [142, 143]. Interestingly, in these latter MCI patients, one study showed that melatonin therapy decreased oxidative stress and increased the activity of AChE [144]. For a list of melatonin doses and models in preclinical studies or of dosage and duration in clinical studies, consult Roy et al. [11] and Tchekalarova and Tzoneva [12].

#### 3.2.2 PD

Melatonin treatment at doses ranging from 5 to 30 mg/kg i.p. alleviated lipid peroxidation, mitochondrial dysfunction, neuroinflammation, and nigrostriatal neurons loss in a variety of in vivo experimental PD models, including mice or rats injected with MPTP [145, 146, 147, 148, 149, 150, 151, 152], 6‐OHDA [153, 154], and rotenone [155]. In several studies, prophylactic melatonin exerted a more pronounced beneficial effect (PD models and doses are reviewed in Tchekalarova and Tzoneva) [12]. Given orally, before kainic acid injection, 50 mg/kg melatonin blocked α‐syn fibril formation in the mouse hippocampus [156]. Interestingly, MT_1_ receptor knockout enhanced α‐syn preformed fibrils‐induced PD‐like pathology in mice and aggravated striatal ferroptosis [157].

Clinically, melatonin treatment improves the quality of sleep in PD (for review, see Ma et al. [158]). In one study, 10 mg/day of melatonin significantly impacted motor symptoms and sleep disturbances in PD patients when immediate‐release formulations were used for ≥ 12 weeks [159]. Nevertheless, the few clinical trials performed to assess the therapeutic potential of melatonin in PD does not allow to make any conclusion on the therapeutic potential of melatonin in PD.

#### 3.2.3 MS

Most of the preclinical studies performed in rodent MS experimental models suggested a positive effect of melatonin on a clinical severity scale. In myelin oligodendrocyte glycoprotein MOG35‐55‐induced EAE, daily melatonin treatment reduced disease severity at onset, peak and chronic stages if given orally (5 mg/kg) or i.p., at both high (10−20−80−200 mg/kg) [70, 160, 161, 162, 163, 164], or low doses (0.1−1 mg/kg, i.p) [165, 166], and significantly prevented demyelination and axonal damage [162, 165]. When started at peak stage, morning melatonin treatment at physiological (476 µg/kg) or pharmacological (10 mg/kg) doses significantly improved functional recovery at chronic EAE stage [70]. Nevertheless, 10 mg/kg melatonin treatment in young EAE rats was not beneficial as resulted in increased demyelination and higher IFN‐γ and lactate serum levels [167]. This negative impact in young rats was restored by inhibiting the pyruvate dehydrogenase complex (PDC) [168].

The effects of melatonin treatment in the cuprizone model were investigated during the demyelination stage. Neither 1 nor 5‐weeks melatonin treatment with high doses (50−80−100 melatonin mg/kg) benefitted myelin damage [169, 170, 171]. Nevertheless, 50−80−100 mg/kg melatonin treatments during demyelination reduced apoptosis, improved locomotor performance in mice, and enhanced the tissue levels of antioxidant enzymes [169, 170, 172]. The effect of melatonin treatments on neuroinflammation during cuprizone‐induced demyelination is unclear. The release of proinflammatory IL‐1β and TNF‐α cytokines was reduced in one study with melatonin 80 mg/kg [169], while Vakilzadeh et al. found upregulated NFκB, IL‐6, and heme oxygenase‐1 protein levels with 50−100 mg/kg melatonin treatment [170]. There is no information on melatonin actions on remyelination rate when it is given after cuprizone withdrawal. Nevertheless, when 80 mg/kg melatonin treatment was started during cuprizone treatment and continued after cuprizone withdrawal, improved locomotor activity and reduced IL‐1β and TNF‐α levels at the end of the remyelination stage were observed (Abo Taleb et al. [169]). Although melatonin may exacerbate some autoimmune conditions [78], it blocks the development of proinflammatory Th17 cells and decreases CNS immune infiltration in MS models [163, 170].

In clinical settings, either short‐ or long‐term melatonin treatment within the range of 3−10 mg increased the plasma antioxidant markers [52], improved sleep, and reduced fatigue scales [173], but did not benefit the disability score (EDSS) both in relapsing‐remitting multiple sclerosis (RRMS) and secondary progressive multiple sclerosis (SPMS) patients [174]. This outcome was neither achieved with 25 mg/kg oral melatonin treatment for 6 months in RRMS patients, although the treatment significantly reduced the serum levels of proinflammatory IL‐1β, IL‐6, and TNF‐α cytokines. Clinical trials are reviewed in Morsali et al. [13]. A case report described an improvement of EDSS scale in a single primary progressive MS (PPMS) patient taking very high doses of melatonin, in the range of 50−300 mg/kg daily [175]. Despite the limited studies on the risk profile of high doses of melatonin in large‐scale cohorts [176], a new clinical trial (NCT03540485) is currently ongoing to evaluate the safety and efficacy of daily administration of 100 mg of melatonin orally for 24 months (single dose of melatonin between 10 p.m. and 11 p.m.) combined with ocrelizumab in patients with PPMS.

## 4 Melatonin Combinations

Melatonin has been evaluated in combination with several molecules claimed as neuroprotective to assess whether the combination could attenuate the progression of neurodegenerative disorders to a greater extent than a single‐agent treatment. Details of in vivo studies are reported in Table 1; a summary of the assays performed for in vitro characterization of melatonin combinations and hybrid derivatives can be found in Table S1.

**Table 1:** Details of in vivo studies performed on melatonin combinations and hybrid molecules.

Combination/hybrid compound	Disease condition or experimental model of disease	Treatment	Outcome	References
Melatonin + memantine	AD model/APP/Ps1 mice	Melatonin: 6 mg/kg/day (po) + memantine: 10 mg/kg/day (po) Duration: 32 days	Improvement in episodic memory and reduction of amyloid aggregates compared to the drugs alone.	[176, 177]
Melatonin + resveratrol	AD model/AlCl_3_ and d‐galactose‐treated mice	Melatonin: 80 mg/kg/day (ip) + resveratrol: 40 mg/kg/day (ip) Duration: 10 weeks	Beneficial effects on recognition memory impairment. No additive or synergistic effect.	[178]
Melatonin + ergothioneine	AD model/d‐galactose‐treated mice	Melatonin: 10 mg/kg/day (po) + ergothioneine: 0.5 mg/kg/day (po) Duration: 88 days	Synergistic neuroprotective effect on learning and memory deficits.	[179]
Melatonin + selegiline	PD model/MPTP‐treated mice	Melatonin: 5/10 mg/kg (po) + selegiline: 0.37 mg/kg (po) Duration: 1 day	Synergistic effect in the recovery of locomotor activity, maintenance of dopamine levels, and tyrosine hydroxylase activity.	[180]
Melatonin + baclofen	MS model/EAE mice	Melatonin: 10 mg/kg/day (ip) + baclofen: 10 mg/kg/day (ip) Duration: 10 days	Improvement in neurological functional recovery, remyelination, and in decreasing oxidative stress and inflammation compared to the drugs alone.	[181]
Melatonin + DADA	MS model/EAE mice	Melatonin: 10 mg/kg/day (po) + DADA: 50 mg/kg/day (po) Duration: 10 days	Reduction of the disability score and proinflammation, and increase of anti‐inflammatory cytokines and myelin‐associated oligodendrocytic basic protein compared to the drugs alone.	[168]
Melatonin + INF‐β	MS model/EAE rats	Melatonin: 20 mg/day (ip) + INF‐β: 9000 UI every other day (ip) Duration: 20 days	Reduction of the disease progression rate and decrease of clinical symptoms, with reduction of plasma proinflammatory cytokines, CNS oxidative stress markers, and brain membrane hyperfluidification. No additive or synergistic effect.	[182]
Melatonin + GA	MS model/EAE rats	Melatonin: 20 mg/day (ip) + GA: 10 mg/day (ip) Duration: 20 days		
**2**	AD model/APP/Ps1 mice	2 µL, 50 µg/mL/day (icv) Duration: 21 days	Reduction of (Aβ)‐induced cell death and amyloid burden in the brain parenchyma and recovery in cognitive function. No comparison with drugs alone or in combination.	[183]
Melatonin‐tacrine hybrid				
**12**	AD model/Aβ_25−35_‐injected mice	0.1 mg/kg/day (ip) Duration: 7 days	Improvements in reduction of Aβ_25−35_‐induced learning deficits and protected long‐term memory compared to the reference BuChE inhibitor.	[184]
Melatonin‐ChE inhibitor hybrid				
**13**	AD model/Aβ_25−35_‐injected mice	0.3 mg/kg/day (ip) Duration: 7 days	Improvements in reduction of Aβ_25−35_‐induced working memory deficits compared to the reference HDAC6 inhibitor and melatonin alone or in combination.	[185]
Melatonin‐ferulic acid‐HDAC inhibitor hybrid				
**16**	AD model/APP/Ps1 mice	50 mg/kg/day (po) Duration: 12 weeks	Improvements in reduction of Aβ brain accumulation and synaptic degeneration, with anti‐inflammatory and antioxidant activities. No comparison with drugs alone or in combination.	[186]
Melatonin‐curcumin hybrid				

### 4.1 AD

Combinations of drugs approved for AD and melatonin have been investigated both in vitro and in vivo. In vitro assays were performed on human SH‐SY5Y neuroblastoma cell line to evaluate whether melatonin in combination with the AChE inhibitor galantamine could protect against mitochondrial oxidative stress induced by rotenone and oligomycin‐A exposure. MTT assay indicated that the combination of sub‐effective concentrations of melatonin (0.3 nM) and galantamine (30 nM, respectively) provided synergistic protection against the oxidative insult. The involvement of nicotinic and melatonin receptors in the neuroprotective mechanism was assessed through pharmacological antagonism studies with mecamylamine and luzindole, respectively, which fully reversed the beneficial effect on the AD model [187].

Similar conclusions were drawn by treating organotypic hippocampal cultures with a combination of subeffective concentrations of melatonin (1 nM) and galantamine (10 nM) in an Aβ/okadaic acid AD model. A reduction of AD‐related pathological hallmarks, Aβ aggregates, and Tau hyperphosphorylation, was observed. Also in this case, nicotinic and melatonin receptor antagonists reversed the positive effects exerted by the drug combination [188]. Analogously, preincubation with a combination of subeffective concentrations of melatonin (1 nM) and donepezil (10 nM) provided significant protection against Aβ/okadaic acid‐induced toxicity in SH‐SY5Y cells compared to the drugs alone [133].

Memantine, a drug approved for AD treatment, has been investigated in combination with melatonin. In APP/PS1 (5xFAD) double transgenic mice, the chronic oral administration of melatonin and memantine (6 and 10 mg/kg, respectively, Table 1) was more effective on memory decline and reduced amyloid aggregates and neuroinflammation with highest efficacy than treatment with melatonin or memantine alone at the same dose used in the combination [177].

Melatonin was also evaluated in combination with antioxidant agents, for example, resveratrol which is able to reduce secreted and intracellular Aβ through proteasome‐dependent Aβ degradation in HEK293 and neuroblastoma N2a cell lines transfected with wild‐type or Swedish mutant APP_695_ (APP_swe_) [189]. In a first study, melatonin and resveratrol, alone or in combination, were tested on Aβ_1−42_‐induced neurotoxicity in HT22 hippocampal cells. All treatments attenuated neuronal cell death, reduced ROS production, restored GSH levels, prevented mitochondrial oxidative stress, and inhibited AMPK phosphorylation. A synergistic effect was observed with subeffective concentrations of melatonin and resveratrol (10 and 1 µM, respectively) [190]. On the other hand, in vivo co‐administration of melatonin and resveratrol (80 and 40 mg/kg, respectively, Table 1) did not show any additive or synergistic prophylactic effect against memory deficits in a non‐transgenic sporadic mouse model of AD [178]. In an in vitro N2a/APP_swe_ cell culture AD model, AD biomarkers were evaluated following treatment with melatonin (30 μM) in combination with naturally occurring compounds resveratrol (10 μM), vitamin B12 (40 μM), and epigallocatechin‐3‐gallate (20 μM). Combinations with two and/or all three substances, but not melatonin alone, were effective in decreasing Aβ levels, ROS production, and release of proinflammatory cytokines and in promoting the expression of antioxidant enzymes [191]. Among other antioxidants, ergothioneine [192] was supplemented with melatonin (0.5 and 10 mg/kg, respectively, Table 1) in the d‐galactose (DG) induction model for AD in C57BL/6 mice, characterized by learning and memory deficits and brain oxidative stress [179]. Both molecules alone improved the behavioral status observed in the water‐maze test and showed antioxidant properties, preventing DG‐induced lipid peroxidation and the decreased GSH/GSSG ratio. Supplementation with melatonin and ergothioneine in combination at the same dose as used alone produced an additive or synergistic neuroprotective effect on learning and memory deficits.

Melatonin has also been evaluated as an add‐on to standard therapy in AD. Patients with MCI treated with melatonin (3−9 mg for 9−18 months) showed ameliorated performance in neuropsychological tests and decreased mood‐related symptoms [142]. Patients with mild to moderate AD receiving prolonged‐released melatonin (2 mg/day for 24 weeks) had ameliorated cognitive performance compared to placebo [141].

### 4.2 PD

PD is a neurodegenerative disease for which a combination of standard treatment (i.e., selegiline) with melatonin has been investigated in vitro and in animal models. Starting from the results of previous studies which demonstrated a potentiated antioxidant effect produced by the combination of the MAO inhibitor selegiline with melatonin in an autoxidation model of dopamine in solution [193], an in vivo PD model, obtained by treating mice with 1‐methyl‐4‐phenyl‐1,2,3,6‐tetrahydropyridine (MPTP), was used to understand the effects of melatonin and selegiline alone and in combination on animal behavior. Simultaneous injection of melatonin and selegiline (5 or 10 mg/kg and 0.37 mg/kg, respectively, Table 1) did not improve the ability of melatonin to reduce the MPTP‐induced lipid peroxidation and mitochondrial damage. On the other hand, co‐administered melatonin potentiated the effects exerted by selegiline alone in the recovery of locomotor activity and maintenance of dopamine levels and tyrosine hydroxylase activity [180].

Combined melatonin treatment with protocatechuic acid [194] and hydroxytyrosol [195], two antioxidants found in fruits and olive oil, respectively, was evaluated for their effects on α‐Syn fibril formation and destabilization, and on vitagenes expression (e.g., SIRT1 and SIRT2). The association of the three molecules (at concentrations ≥ 25 μM) was the most effective against α‐Syn fibril formation, but melatonin did not reinforce the destabilization of pre‐formed α‐Syn fibrils produced by the other two substances. The triple combination (25, 10, and 10 μM, respectively) was the most effective in protecting PC12 cells from α‐Syn‐induced toxicity and increased the expression of SIRT2 in the absence of α‐Syn fibrils, highlighting the preventive potential of the combination and suggesting that attention should be given to diet composition to delay neurodegenerative processes [196].

### 4.3 MS

An in vivo study conducted on an EAE mouse model of MS demonstrated that addition of baclofen, a muscle relaxant drug, to melatonin (both molecules at 10 mg/kg, Table 1) improved neurological functional recovery compared to melatonin alone and enhanced the efficiency of melatonin in promoting remyelination and in decreasing oxidative stress and inflammation [181].

Melatonin treatment of EAE mice increases brain expression of pyruvate dehydrogenase kinase‐4 responsible for the inhibition of PDC, a key enzyme in fatty acid synthesis during the remyelination process, likely affecting the efficiency of treatment [70]. On this basis, combination of the PDK4 inhibitor diisopropylamine dichloroacetate (DADA) and melatonin (50 and 10 mg/kg, respectively, Table 1) was evaluated in the EAE model, aiming at the minimization of melatonin side effects. Combination therapy decreased the disability score, reduced levels of proinflammatory and increased anti‐inflammatory cytokines and myelin‐associated oligodendrocytic basic protein to a greater extent than melatonin alone, restoring PDC activity [168]. In another study, a combination of melatonin (20 mg/day) and interferon beta (INF‐β, 9000 IU every other day) or glatiramer acetate (GA, 10 mg/day) slowed down disease progression rate and decreased clinical symptoms in EAE rats (Table 1). Additionally, a reduction of plasma proinflammatory cytokines, CNS oxidative stress markers, and brain membrane hyperfluidification was observed. However, the effects obtained for combination treatments were not greater than those observed for melatonin alone [182].

The efficacy of melatonin as an add‐on to standard therapy was evaluated in MS patients. The field has been recently reviewed [13]. Melatonin supplementation was generally related to reduced oxidative stress markers and levels of proinflammatory cytokines, and higher activity of antioxidant enzymes. Decreased fatigue scores and improved quality of life were also recorded. The reasons for the failure to achieve these outcomes in some studies might be related to the variable length of treatment (2 weeks to 1 year) and dose administered (0.5−25 mg), as well as to the disease subtypes considered (RRMS, SPMS, and PPMS). A clinical trial to evaluate the safety and efficacy of melatonin (100 mg/day) administration combined with ocrelizumab in patients with progressive multiple primary sclerosis is currently ongoing (NCT03540485).

Overall, the field appears highly varied, both in terms of choice of cellular and animal models and of doses investigated. In vitro, melatonin was evaluated at nanomolar concentrations when combined with approved drugs (e.g., galantamine) [187, 188], but at much higher micromolar concentrations when tested in combination with antioxidant agents (e.g., resveratrol) [190]. As discussed for experiments performed with melatonin alone, results from investigations at pharmacological concentrations pose serious issues about their translational potential, in terms of administered doses and concentrations reached in vivo, mechanisms, and toxicity, with no real significance and advantage in terms of human health [19, 118]. The mechanisms affecting the results obtained at high pharmacological concentrations could be different from those operating at much lower concentrations and might affect the outcomes observed for the combination and the generation of additive or synergistic effects.

Several doses, different routes of administration, and lengths of treatment were employed in in vivo studies, with, for example, 5−80 mg/kg of melatonin administered to mice (Table 1). This heterogeneity is likely the reason for the different results recorded, as synergistic effects or no improvement compared to single‐agent treatment were both reported. The same heterogeneity of treatment conditions and outcomes affects studies performed on patients assuming melatonin as an add‐on to their standard therapy.

## 5 Melatonin Hybrids

Several melatonin‐containing hybrid molecules were investigated as new options for the treatment of neurodegenerative disorders. Most of these molecules were tested in experimental models of AD [11] due to its multifactorial nature and related pathophysiological changes that make the single‐target therapeutic approach inadequate for disease treatment [197]. However, given the common pathological processes contributing to neuronal dysfunction, including glutamate excitotoxicity, mitochondrial dysfunction, and free radical‐mediated damage, other neurodegenerative disorders may benefit from the multitarget‐directed approach [198]. The strategy pursued in the design of multitarget compounds generally relied on the exploitation of the antioxidant properties of melatonin that was combined with other antioxidant agents or with compounds with different, potentially synergistic activities. Herein, the structure and rationale of melatonin hybrids is described, with the reported in vitro and/or in vivo pharmacological characterization. In light of the potential receptor‐mediated activity, we focused on compounds characterized by the indolyl‐ethylamide structure of melatonin. On the other hand, multitarget indole derivatives devoid of the ethylamide side chain and connected to molecular portions from other neuroprotective agents (e.g., trientine [199], donepezil [200, 201, 202, 203], sulforaphane [204, 205], have also been described.

**Figure 1:**
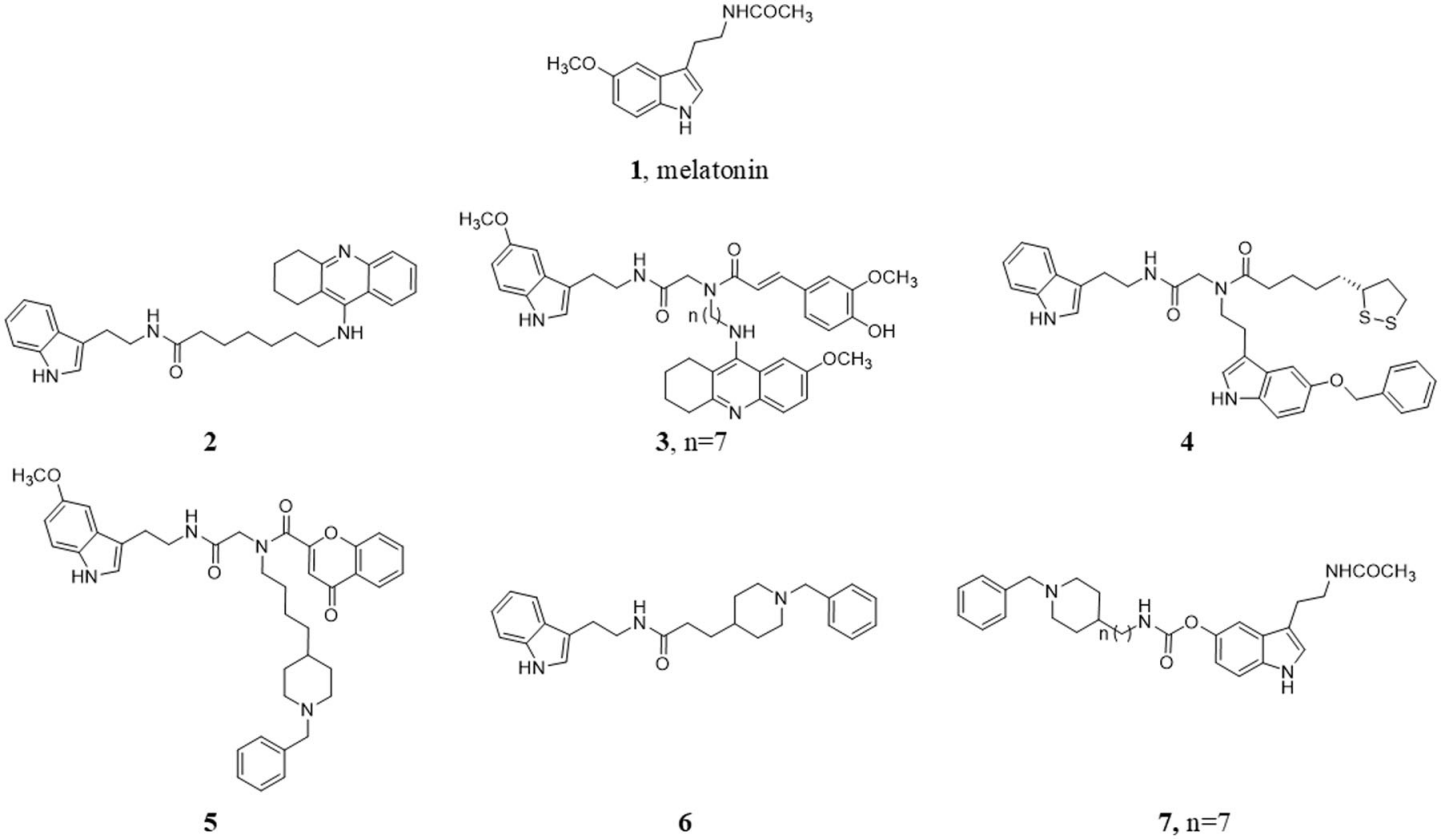
Chemical structures of melatonin (**1**) and melatonin‐containing hybrids **2**−**7**.

The first reported melatonin‐containing hybrid molecules were designed to combine the free‐radical scavenging and antioxidant properties of melatonin **1** (Figure 1) with the anticholinesterase activity of tacrine. The amino group of tacrine was connected to the amide side chain of melatonin (e.g., compound **2**, Figure 1) which represents the preferred site for melatonin modification in hybrid molecules [206, 207]. Compared to tacrine and melatonin, hybrid **2** exhibited improved in vitro properties, that is, higher AChE and BuChE inhibition and oxygen radical absorbance capacity (ORAC assay). Compounds from this series also inhibited Aβ self‐aggregation and exerted neuroprotective properties in human neuroblastoma cell line SH‐SY5Y against cell death induced by various toxic insults [208]. Furthermore, compound **2** produced a significant decrease of Aβ deposits with a reduction of Aβ‐induced cell death in organotypic slice cultures and alleviation of behavioral impairment, with recovery in memory and cognitive function, in amyloid precursor protein/presenilin 1 (APP/Ps1) transgenic mice (2 µL, 50 µg/mL, Table 1). No comparison with melatonin efficacy was made [183]. Another melatonin‐tacrine hybrid was based on the addition of ferulic acid as an adjunct antioxidant element. Compound **3** (Figure 1) showed an enhanced antioxidant profile (ORAC assay) compared to the parent compounds, suggesting a synergistic effect between the components of the hybrid molecule. Compound **3** displayed higher activation of the Nrf2 transcriptional pathway than melatonin and neuroprotective activity against Aβ peptides and other toxic stimuli in SH‐SY5Y cells [209].

Following the same antioxidant additive approach, two melatonin molecules were linked to the antioxidants ferulic acid, lipoic acid, or comenic acid. The lipoic acid derivative **4** (Figure 1) exhibited improved antioxidant activity compared to melatonin (ORAC assay), potently induced Nrf2 transcriptional pathway, being approximately twofold more potent than melatonin, and exerted neuroprotective activity on SH‐SY5Y cell line counteracting H_2_O_2_ cytotoxic insult [210].

Besides tacrine, other AD anticholinesterase drugs were exploited for the design of hybrid derivatives. The benzylpiperidine moiety of donepezil was incorporated into the side chain of melatonin together with a 2‐chromone portion. The 2‐chromone ring is the core fragment of several monoamine oxidase (MAO) inhibitors, which reduce the formation of neurotoxic H_2_O_2_ and ROS species [211]. Hybrid compound **5** (Figure 1) was characterized by moderate cholinesterase and MAO inhibitory activity and showed antioxidant properties comparable to those of melatonin (ORAC assay). In another series of multifunctional molecules, the benzylpiperidine moiety of donepezil was joined to the amide side chain of melatonin either directly or through alkyl spacers [212]. Hybrid compound **6** (Figure 1) showed reversed cholinesterase selectivity compared to the parent donepezil, being more potent against BuChE than AChE, greater ROS scavenging ability than melatonin (ORAC assay), chelating ability, moderate inhibition of Aβ_1−42_ self‐aggregation, and higher neuroprotective effect against H_2_O_2_‐induced cell death on PC12 cell line than melatonin. The benzylpiperidine fragment was also linked to position 5 of the indole ring of melatonin via a carbamate group, as in compound **7** (Figure 1) which showed improved selectivity toward BuChE, expressed at high levels in advanced AD [213].

**Figure 2:**
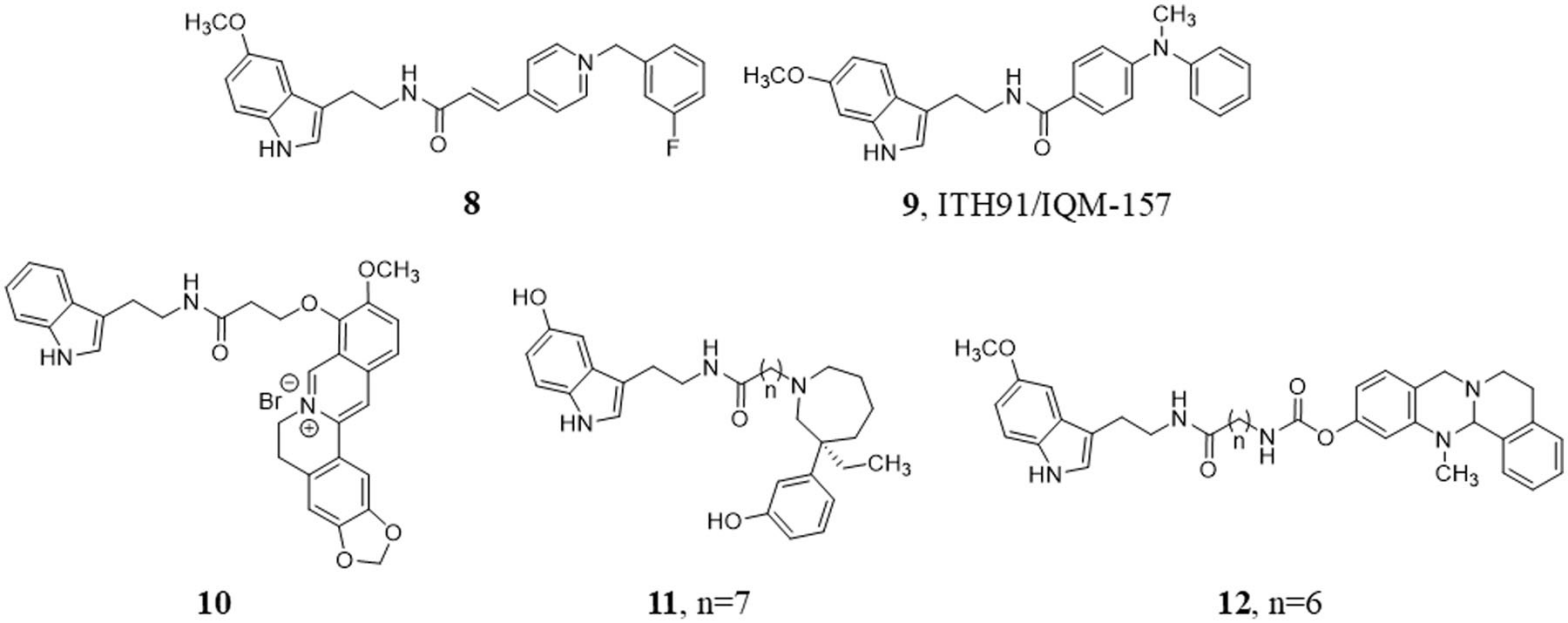
Chemical structures of melatonin‐containing hybrids **8**−**12**.

Another anticholinesterase scaffold exploited for the design of hybrid compounds was benzylpyridinium bromide, which was installed on melatonin side chain giving **8** (Figure 2), a potent *h*AChE and *h*BuChE inhibitor with greater antioxidant activity than melatonin (ORAC assay) and neuroprotective effect against H_2_O_2_‐induced damage in neuroblastoma SH‐SY5Y cell line comparable to that of melatonin [214].

The *N,N*‑dibenzyl(*N*‑methyl)amine fragment of the AChE inhibitor AP2238 [215] was inserted on the amide side chain of melatonin, looking for hybrid compounds with anticholinesterase, antioxidant, neuroprotective, and neurogenic effects. These compounds had reduced AChE and BuChE inhibitory activity compared to tacrine and antioxidant activity in the same range as melatonin (ORAC assay). They also showed good levels of neuroprotection in SH‐SY5Y neuroblastoma cell line and were more potent than melatonin to stimulate early neurogenesis and cell maturation into a neuronal phenotype of rat hippocampal primary neuronal stem cells [216]. ITH91/IQM‐157 (**9**, Figure 2) exerted neuroprotection in SH‐SY5Y cells in which cytotoxicity was induced by a combination of Aβ and okadaic acid to reproduce markers of both beta and tau pathologies. It demonstrated greater neuroprotective activity than the combination of subeffective concentrations of melatonin and donepezil. Melatonin receptors appear to be involved in the neuroprotective mechanism since the antagonist luzindole significantly reduced the protective effect [133].

Berberine and (−)‐meptazinol are other cholinesterase inhibitors linked to the amide side chain of melatonin to combine enzyme inhibition with the antioxidant and anti‐Aβ‐aggregation activities of melatonin [217, 218]. Berberine derivative **10** (Figure 2) showed improved antioxidant activity compared to melatonin and a greater ability to prevent Aβ aggregation than berberine. Similarly, hybrid **11** (Figure 2) displayed higher inhibition of AChE than (−)‐meptazinol and of Aβ_1−42_ self‐aggregation than curcumin.

Another interesting hybrid derivative was based on functionalization of melatonin amide side chain with a pseudo‐irreversible carbamate BuChE inhibitor, selective over AChE which is less involved in the later stages of AD [184]. Insertion of a six‐methylene linker led to compound **12** (Figure 2) with reduced potency toward BuChE but increased duration of action, significantly protecting murine hippocampal neuronal HT22 cells from glutamate neurotoxicity. Compound **12** reduced LPS‐induced microglia inflammation and exhibited immunomodulatory activity, promoting a switch from neurotoxic M1 to neuroprotective M2 phenotype in N9 microglial cells, with improved activity compared to melatonin. Compound **12** reduced Aβ_25−35_‐induced learning deficits and protected long‐term memory in mice at very low dosage (0.1 mg/kg, Table 1), with significantly higher in vivo efficacy than the parent BuChE inhibitor [184].

**Figure 3:**
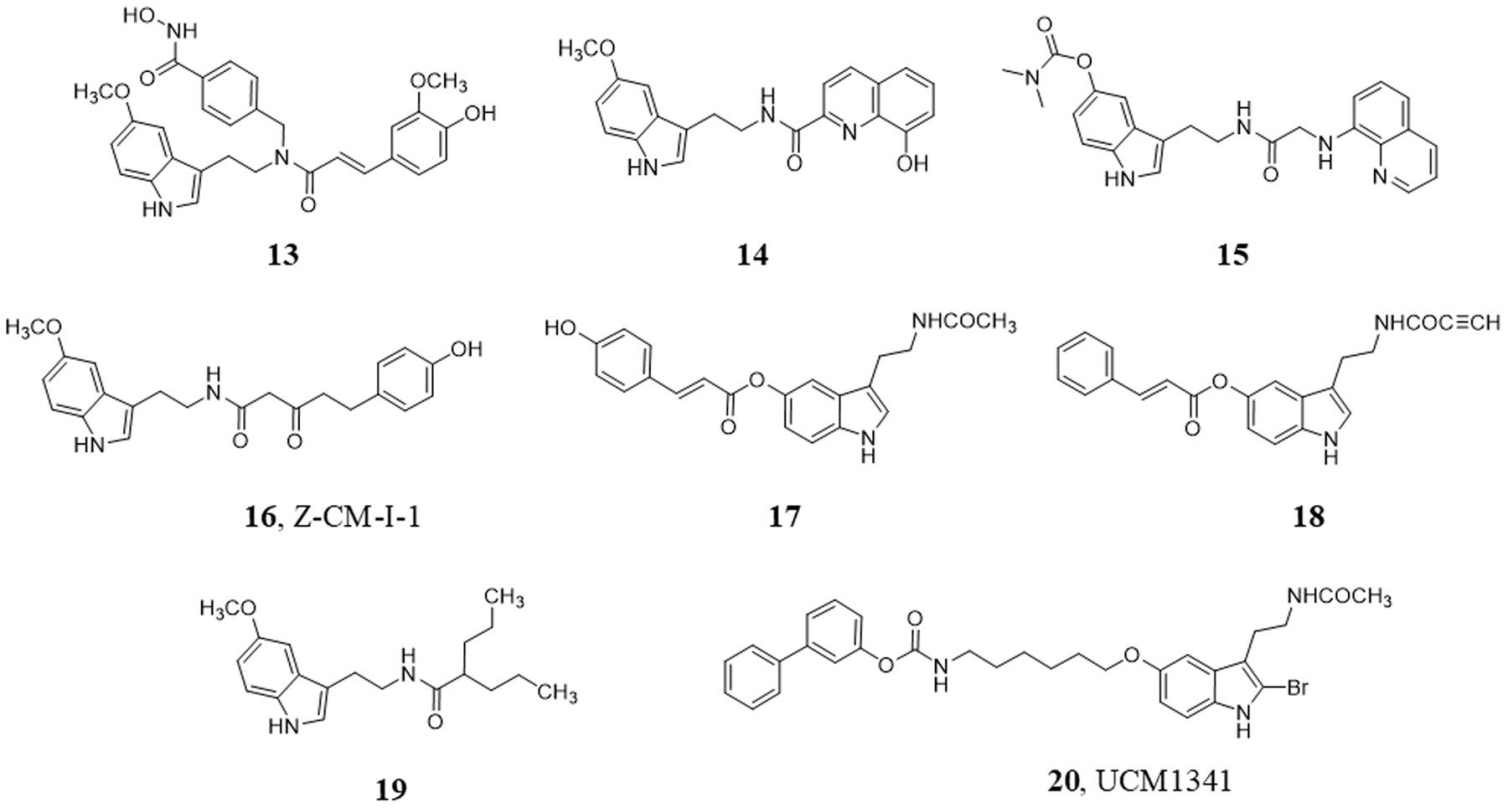
Chemical structures of melatonin‐containing hybrids **13**−**20**.

The multitarget‐directed ligand **13** (Figure 3) was obtained, merging melatonin and ferulic acid into the structure of a selective phenylhydroxamic acid‐based HDAC6 inhibitor. Compound **13** was investigated as a new drug candidate for AD disease [185]. It maintained activity and selectivity toward HDAC6 and showed chelating ability and antioxidant effects with efficacy comparable to those of melatonin and ferulic acid. Compound **13** showed immunomodulatory activity, reducing LPS‐induced inflammation of microglia and promoting a shift from M1 to M2 phenotype of N9 microglial cells. The neuroprotective activity of compound **13** was assayed in a mouse model of AD (Table 1), where it attenuated Aβ_25−35_‐induced working memory deficits at a lower dose (0.3 mg/kg) than the reference HDAC6 inhibitor (1 mg/kg). In the same experiment, melatonin (0.16 mg/kg) and an equimolar (0.7 μM) mixture of the three parent compounds were ineffective.

Following a similar strategy, the amide chain of melatonin was fused with 8‐hydroxyquinoline, an ion‐chelating agent, resulting in melatonin hybrids with greater antioxidant capacity and inhibition of Aβ_1−42_ aggregation compared to melatonin and clioquinol [219]. Compound **14** (Figure 3) alleviated H_2_O_2_‐induced oxidative stress in SH‐SYSH cells and disaggregated Cu2+‐induced Aβ_1−42_ aggregation with an effect superior to that of melatonin.

The inclusion of a chelating portion and a cholinesterase‐inhibiting carbamate on melatonin structure was investigated to obtain a “multi‐target, multifunctional” synergistic effect for the treatment of AD, leading to compounds exemplified by the 8‐aminoquinoline derivative **15** (Figure 3). However, the most protective compounds on HT22 cell line were the phenolic precursors, devoid of the carbamate group [220].

To enhance the efficacy of melatonin, hybrid compounds with other antioxidant agents were also devised. Incorporation of the β‐diketone moiety of curcumin resulted in compounds represented by derivative Z‐CM‐I‐1 (**16**, Figure 3) which showed neuroprotection in a cellular AD model (MC65 cells that conditionally overexpress the 99‐residue carboxyl‐terminal [C99] fragment of the amyloid precursor protein) [221]. Interestingly, the hybrid compound was protective at 0.3 µM concentration, at which melatonin, curcumin, and their combination showed no effect. The neuroprotective activity was mainly related to the potent intracellular antioxidant effect, as hybrid **16** reduced the production of Aβ oligomers with low potency and did not inhibit Aβ_42_ fibrillization. In vivo studies demonstrated that compound **16** given orally at doses of 50 mg/kg for 12 weeks (Table 1) decreased Aβ brain accumulation, exerted anti‐inflammatory and antioxidant activities in APP/PS1 mice, and significantly reduced synaptic degeneration. These multiple effects sustain its therapeutic potential on memory and cognitive deficits in AD [186].

Since Nrf‐2 activation is known to regulate antioxidant and antinflammatory responses, melatonin scaffold was decorated with potent Nrf‐2 inducers to obtain hybrid compounds with multi‐target activity for the treatment of neurodegenerative diseases. Compound **17** (Figure 3), obtained by merging an ethyl cinnamate derivative in position 5 of the indole nucleus of melatonin, exhibited grater Nrf2 induction activity and antioxidant properties (ORAC assay) than both precursor molecules. This hybrid derivative exerted neuroprotective activity in two models of oxidative stress in SH‐SY5Y neuroblastoma cells and in oxygen and glucose deprivation models in rat hippocampal slices, with higher efficacy than melatonin [222]. Within a similar multitarget strategy, compound **18** (Figure 3) showed antioxidant properties and Nrf2‐inducing activity. It exerted a neuroprotective activity against toxic stimuli in SH‐SY5Y cells comparable to that of reference compounds melatonin and rasagiline [223].

Melatonylvalpromide (**19**, Figure 3) is a hybrid molecule deriving from the combination of melatonin and valproic acid as the acylating group in the amide side chain of melatonin. Valproic acid upregulates melatonin receptors and neurotrophic factors. When tested in neuroblastoma 2a cells stably transfected with human APP695 (N2a/APP), it preserved cell viability, led to a greater reduction of Aβ levels in cell lysates than melatonin at the same concentration (10 µM), and promoted dephosphorylation of neurofilament proteins [224].

The dual‐acting compound UCM1341 (**20**, Figure 3) [225, 226] combines fatty acid amide hydrolase (FAAH) inhibitory activity with agonist activity at melatonin receptors. At 10 µM concentration, it exhibited greater neuroprotection against a neuroinflammatory insult (INFγ + LPS) in hippocampal slice cultures than the reference FAAH inhibitor URB597 [227] and melatonin alone at the same concentration, and comparable to that of the combination of the two agents. Counteracting neuroinflammation, compound **20** enhanced the levels of anandamide and oleoylethanolamide, prevented TNF‐α release, and upregulated the expression of PPARα. The neuroprotective effects of **20** were prevented by PPARα, TRPV1, and melatonin receptor antagonists, confirming a receptor‐dependent mechanism for the melatonergic component.

Overall, melatonin was merged with (or with a portion of) neuroprotective agents acting according to a variety of mechanisms, spanning from enzyme inhibition (e.g., cholinesterase, HDAC, and FAAH) to metal chelation and antioxidant activity. The efficacy of the melatonergic component was mainly related to the antioxidant effect, with increased transcription of antioxidant enzymes and modulation of pro‐ and anti‐inflammatory cytokines. On the other hand, the involvement of melatonin receptors and other cellular targets [25] was poorly investigated. In fact, despite the consolidated role of receptor engagement in the neuroprotective activity of melatonin, very few hybrid molecules (**9** and **20**) were assayed for their affinity and behavior at MT_1_ and MT_2_ receptors, limiting the characterization of their molecular mechanism, which should represent an indispensable requisite for the rational combination of multiple activities. Additionally, once verified the role of receptor engagement, the development of new agents could be pushed further according to known structure–activity relationships for melatonergic ligands. In this line, replacement of the ever‐present indole portion with suitable bioisosteres might provide a selective modulation of receptor activity (e.g., MT_1_ vs. MT_2_ engagement) and structure optimization [228, 229]. In fact, hybrid compounds described so far appear more as proof‐of‐principle pharmacological tools than optimized drug candidates, with in vivo evaluation limited to few compounds and no investigation of biopharmaceutical properties and pharmacokinetic profile.

The 5‐methoxy group of melatonin is important, albeit not strictly necessary, for agonist activity at melatonin receptors. On the other hand, it is amenable to metabolic demethylation, and this can be regarded as a general liability for aromatic methoxyl derivatives. In fact, several works [183, 207, 210, 212] had shown that compounds lacking a 5‐methoxy group, such as in the *N*‐acetyltryptamine fragment, can provide interesting combined effects in neuroprotective assays. Comparing the activities reported for couples of unsubstituted and methoxy‐substituted hybrids, a very limited effect can be ascribed to the methoxy group which generally produces a slight increase in the antioxidant activity measured in the ORAC assay and a moderate reduction of cholinesterase inhibitory activity. The effect of the methoxy group on Aβ peptide aggregation inhibition is series‐dependent.

The characterization of mechanisms and interpretation of activity of hybrid compounds is complicated by the choice of the cell models used for compound evaluation since the expression of melatonin receptors in cells used for assays is taken into consideration only for compounds **9** and **20**. The better performance of hybrid compounds compared to melatonin might be due to an effective synergistic activity, but also to a difference in the expression of the targets of melatonin and the combined agent. Additionally, the superior behavior of hybrid compounds versus the combination of single drugs still needs to be verified, both in animal models and in a clinical setting.

## 6 Conclusions

The multifactorial pathogenesis of AD, PD, and MS has prompted the investigation of drug combinations and hybrid compounds with multiple mechanisms as disease‐modifying treatments. In this context, melatonin has been evaluated in combination with approved or investigational drugs and with natural substances. It has also been included in hybrid molecules, fused with compounds exerting similar activities (e.g., antioxidant, Nrf2 inducer) or with no overlapping mechanisms. In the latter case, different signaling pathways are activated, even if the same target can be affected by both merged portions, as observed for melatonin fused with acetylcholinesterase inhibitors binding the peripheric anionic site and the catalytic site, respectively.

Overall, most combinations and hybrid molecules produced an improvement in disease‐related biological markers and animal conditions compared to single‐agent treatment, with synergistic effects recorded for combinations with both approved drugs and natural compounds. On the patient side, melatonin adjunct to standard therapy often improved quality of life, which can be an indirect effect of sleep regulation, even if a clinical benefit was also recorded in some cases. In fact, melatonin has been proposed as a safe auxiliary medication to manage disease symptoms in MS patients [230]. On the other hand, it is not possible to draw conclusions on the generalizability and translational potential of these melatonin‐based therapies, as both combinations and hybrid molecules have been investigated in very different conditions, in terms of cellular and animal models, of patient disease gravity, dose and treatment length and recorded outputs. This heterogeneity of experimental conditions is likely one of the reasons for the diverse results reported in the literature, with promising synergistic effects, additive effects, but also with no improvements compared to single drug treatment. The positive outcomes obtained in vitro need to be challenged in more complex models as they may not translate into in vivo efficacy, as observed in the case of melatonin and resveratrol combination [178, 190]. A relevant issue is posed by the high, supra‐physiological concentrations used in experiments performed in vitro that cannot be reached in vivo and limit the value and the usefulness of the results thus observed.

Another issue requiring further investigation is the potential toxicity of melatonin combinations. The noxious effects produced by the co‐administration of melatonin with other agents have to be carefully checked, in particular in the perspective of a chronic treatment of patients with multiple pathologies, as it is often the case of elderly people.

The case of agomelatine, the hybrid melatonin receptor agonist‐5HT_2C_ receptor antagonist used for the treatment of major depression [231] showed that combined mechanisms can be exploited for therapeutical applications that cannot be afforded by melatonergic agents alone. In the field of neurodegenerative diseases, the use of melatonin in clinical protocols for the treatment of AD and MS (see above) reveals the interest of developing new hybrids which can provide the neuroprotective and sleep‐promoting activities of melatonergic agents combined with other favorable mechanisms. This approach is particularly intriguing when some form of synergism is shown for the two mechanisms, such as in the case of melatonin receptor agonists‐FAAH inhibitors, where melatonin receptor activation stimulates the expression of PPAR, and FAAH inhibition enhances PPAR activation [226]. Even if no hybrid compound is still in the clinical phases for neurodegenerative disease, it is important that precompetitive research provides new pharmacological tools to investigate the therapeutical potential of combined approaches.

## Author Contributions

All authors participated in the writing of the first draft and in the editing of the final version of the review article.

## Conflicts of Interest

The authors declare no conflicts of interest.

## Supporting information

Supporting information.

## Data Availability

Data sharing is not applicable to this article as no new data sets were generated during the current study.
