# Supplementary material for: Protective Activity of Melatonin Combinations and Melatonin‐Based Hybrid Molecules in Neurodegenerative Diseases
Source: J Pineal Res. 2024 Nov 25;76(8):e70008. doi: 10.1111/jpi.70008 (PMC11586835; doi:10.1111/jpi.70008)
Supplement: Supplementary file 1 — Supporting information. [file JPI-76-e70008-s001.PDF]

Protective activity of melatonin combinations and melatonin-based hybrid molecules in neurodegenerative diseases.

Francesca Galvani,[1] Mariarosaria Cammarota,[2] Federica Vacondio,[1] Silvia Rivara,[1,*] Francesca Boscia[2]

[1]Department of Food and Drug, University of Parma, Parma, Italy.

[2]Division of Pharmacology, Department of Neuroscience, Reproductive Sciences and Dentistry, School of Medicine, Federico II University of Naples, Naples, Italy.

SUPPORTING INFORMATION

Table S1. Details of *in vitro* characterization of melatonin combinations and hybrid derivatives. Toxic insult, drug concentration and incubation time are reported for neuroprotection experiments.[a]

Combination/ hybrid compound	Enzyme inhib.	Aβ–self aggregation inhib.[b]	Antiox. activity[c]	Metal chelation	Nrf2 induction	Neuroprotection in cells	Other assays	Ref.
Melatonin + donepezil						*Aβ/oligomycin A-induced toxicity*<br>melatonin: 1 nM, donepezil: 10 nM<br>melatonin + donepezil: 1 + 10 nM<br>incub. time: 24 h pre-treatment + 20 h assay		133
Melatonin + galantamine						*Rotenone/oligomycin A-induced toxicity*<br>melatonin: 0.3 – 10 nM, galantamine: 30 – 300 nM<br>melatonin + galantamine: 0.3 + 30 nM<br>incub. time: 24 h pre-treatment + 24 h assay	A	187
		✓*				*Aβ/oligomycin A-induced toxicity*<br>melatonin: 1 – 1000 nM, galantamine: 10 – 1000 nM<br>melatonin + galantamine: 1 + 10 nM<br>incub. time: 4 days	B	188
Melatonin + resveratrol			✓*			*Aβ-induced toxicity*<br>melatonin: 1 – 500 μM, resveratrol: 0.1 – 20 μM<br>melatonin + resveratrol: 1 + 0.1 μM or 1 + 10 μM<br>incub. time: 24 – 48 h	C	190
Melatonin + protocatechuic ac. + hydroxytyrosol						*α-Syn-induced toxicity*<br>melatonin: 25 μM, protocatechuic ac.: 10 μM, hydroxytyrosol: 10 μM<br>melatonin + protocatechuic ac. + hydroxytyrosol: 25 + 10 + 10 μM<br>incub. time: 24 h	D	196
**2**<br>Melatonin-tacrine hybrid	ChE		✓					206
	ChE	✓	✓			*Aβ-induced toxicity*<br>**2**: 0.1 nM – 10 μM<br>incub. time: 24 h<br><br>*H_2O_2-induced toxicity*<br>**2**: 0.3 μM – 3 μM<br>incub. time: 24 h	E	208
**3**<br>Melatonin-tacrine-ferulic acid hybrid	ChE		✓		✓	*Aβ- or H_2O_2- or rotenone/oligomycin A-induced toxicity*<br>**3**: 1 – 3 μM<br>melatonin: 0.01 – 3 μM, methyl ferulate: 1 – 3 μM<br>incub. time: 24 h		209
**4**<br>Melatonin-ferulic acid hybrid			✓		✓	*H_2O_2-induced toxicity*<br>**4**: 1 – 10 μM<br>melatonin: 1 μM<br>incub. time: 24 h pre-treatment + 24 h assay		210

Combination/ hybrid compound	Enzyme inhib.	Aβ–self aggregation inhib.[b]	Antiox. activity[c]	Metal chelation	Nrf2 induction	Neuroprotection in cells	Other assays	Ref.
**5**<br>Melatonin-donepezil-MAO inhibitor hybrid	ChE<br>MAO		✓					211
**6**<br>Melatonin-donepezil hybrid	ChE	✓	✓	✓		*H_2O_2-induced toxicity*<br>**6**: 1.25 – 10 µM, 24 h<br>melatonin: 10 µM<br>incub. time: 24 h		212
**7**<br>Melatonin-donepezil hybrid	ChE							213
**8**<br>Melatonin-benzylpyridinium bromide hybrid	ChE		✓			*H_2O_2-induced toxicity*<br>**8**: 1 – 10 µM<br>melatonin: 10 µM<br>incub. time: 24 h		214
**9**<br>Melatonin-AP2238 hybrid						*Aβ/oligomycin A-induced toxicity*<br>**9**: 1 – 1000 nM<br>melatonin: 1 – 10 nM, donepezil: 300 – 3000 nM<br>melatonin + donepezil: 1 nM + 10 nM<br>incub. time: 24 h<br>incub. time: 24 h pre-treatment + 20 h assay	F	133
	ChE		✓			*Aβ/oligomycin A-induced toxicity*<br>**10**: 1 µM<br>melatonin: 1 µM<br>incub. time: 24 h pre-treatment + 24 h assay	G	216
**10**<br>Melatonin-berberine hybrid	ChE	✓	✓					217
**11**<br>Melatonin-(-)meptazinol hybrid	ChE	✓	✓					218
**12**<br>Melatonin-ChE inhibitor hybrid	ChE		✓			*Glutamate-induced toxicity*<br>**12**: 1 – 25 µM<br>melatonin: 1 – 25 µM<br>24 h pre-treatment + 12 h assay	H	184

Combination/ hybrid compound	Enzyme inhib.	Aβ–self aggregation inhib.[b]	Antiox. activity[c]	Metal chelation	Nrf2 induction	Neuroprotection in cells	Other assays	Ref.
**13** Melatonin-ferulic acid-HDAC inhibitor	HDAC		✓	✓			I	185
**14** Melatonin-8-hydroxyquinoline hybrid		✓	✓	✓		*H_2O_2-induced toxicity*<br>**14**: 1 – 5 µM		219
**15** Melatonin-8-aminoquinoline hybrid	ChE	✓		✓		*Glutamate-induced toxicity*<br>**15**: 3 – 30 µM<br>melatonin: 3 – 30 µM<br>30 min pre-treatment + 24 h assay		220
**16** Melatonin-curcumin hybrid		✓	✓*			*Tetracycline deprivation-induced toxicity*<br>**16**: 0.1 – 1 µM<br>72 h pre-treatment + 4h assay	J	221
**17** Melatonin-ethyl cinnamate hybrid			✓		✓	*Rotenone/oligomycin A-induced toxicity*<br>**17**: 1 µM<br>melatonin: 1 µM<br>24 h pre-treatment + 24h assay<br><br>*okadaic acid-induced toxicity*<br>**17**: 0.3 – 3 µM<br>melatonin: 3 µM<br>incub. time: 24 h<br><br>*oxygen/glucose deprivation-induced toxicity*<br>**17**: 0.3 – 3 µM<br>melatonin: 3 µM<br>incub. time: 15 min + 2h re-oxygenation		222
**18** Melatonin-ethyl cinnamate hybrid	MAO		✓		✓	*Rotenone- or 6-hydroxydopamine-induced toxicity*<br>**18**: 0.1 µM<br>melatonin: 0.1 µM, rasagiline: 0.1 µM<br>incub. time: 24 h pre-treatment + 24 h assay	K	223
**19** Melatonin-valproic acid hybrid						*Amyloid precursor protein-induced toxicity*<br>**19**: 5 – 50 µM<br>melatonin: 5 – 50 µM<br>incub. time: 12 h	L	224

Combination/ hybrid compound	Enzyme inhib.	Aβ–self aggregation inhib.[b]	Antiox. activity[c]	Metal chelation	Nrf2 induction	Neuroprotection in cells	Other assays	Ref.
**20**<br>Melatonin-URB597 hybrid						*NMDA-induced toxicity or LPS + INF-γ- induced toxicity*<br>**20**: 0.1 – 10 μM<br>melatonin: 10 μM, URB597: 10 μM<br>melatonin + URB597: 10 μM + 10 μM<br>incub. time: 48 – 96 h	M	226

[a] Symbol ✓ indicates assay performed for a specific combination or hybrid compound. [b] Symbol * refers to assays performed in cells. [c] Antioxidant activity was evaluated in acellular assays (e.g., ORAC and DPPH; symbol * refers to antioxidant activity assays performed in cells. A) Involvement of melatonin and nicotinic receptors in the mechanism of action. B) Involvement of melatonin and AChE in the mechanism of action; C) Intracellular GSH levels; $A\beta_{1-42}$-induced MAPK signaling, GSK3β and AMPK signal activation. D) Inhibition of α-Syn fibril formation and destabilization of α-Syn fibrils; expression of sirtuin-1 (SIRT-1), sirtuin-2 (SIRT-2), heat shock protein 70 (Hsp70) and heme oxygenase (HO-1). E) Displacement of propidium iodide from PAS. F) Involvement of melatonin and nicotinic receptors, protein kinase C, ERK 1/2, Akt and SnPP in the mechanism of action; expression of ERK 1/2, Akt and HO-1. G) Displacement of propidium iodide from PAS; evaluation of neurogenic activity. H) Immunomodulatory activity. I) Immunomodulatory activity; expression of acetylated tubulin, acetylated histone H3/4, and Hsp90. J) Inhibition of ROS production in mitochondria. K) reduction of nitrite production (EC_{50} values); reduction of IL-1β levels; quantification of Nrf2-dependent proteins. L) Immunocytochemical studies on neurofilament proteins. M) Involvement of melatonin and nicotinic receptors; GSH levels in hippocampal cultures; quantification of IL-6, IL-10 and TNF-α; expression of mannose receptor CD206, arginase-1, AMPK.
